# Seasonal resource partitioning between neighboring cougars in a multiprey system

**DOI:** 10.1093/beheco/arag060

**Published:** 2026-06-04

**Authors:** Siobhan Darlington, Karen E Hodges, T J Gooliaff, Samuel T Foster, Michael J Noonan, Adam T Ford

**Affiliations:** Department of Biology, The University of British Columbia Okanagan, 1177 Research Road, Kelowna, British Columbia V1V 1V7, Canada; Ministry of Water, Land and Resource Stewardship, Government of British Columbia, 102 Industrial Pl., Penticton, British Columbia V2A 7C8, Canada; Department of Biology, The University of British Columbia Okanagan, 1177 Research Road, Kelowna, British Columbia V1V 1V7, Canada; Okanagan Institute for Biodiversity, Resilience, and Ecosystem Services, The University of British Columbia Okanagan, 1177 Research Road, Kelowna, British Columbia V1V 1V7, Canada; Ministry of Water, Land and Resource Stewardship, Government of British Columbia, 102 Industrial Pl., Penticton, British Columbia V2A 7C8, Canada; Department of Fish and Wildlife Resources, University of Idaho, 875 Perimeter Drive MS1136, Moscow, Idaho 83844-1136, United States; Ministry of Water, Land and Resource Stewardship, Government of British Columbia, #401 333 Victoria St.,Nelson, British Columbia V1L 4K3, Canada; Department of Biology, The University of British Columbia Okanagan, 1177 Research Road, Kelowna, British Columbia V1V 1V7, Canada; Okanagan Institute for Biodiversity, Resilience, and Ecosystem Services, The University of British Columbia Okanagan, 1177 Research Road, Kelowna, British Columbia V1V 1V7, Canada; Department of Biology, The University of British Columbia Okanagan, 1177 Research Road, Kelowna, British Columbia V1V 1V7, Canada; Okanagan Institute for Biodiversity, Resilience, and Ecosystem Services, The University of British Columbia Okanagan, 1177 Research Road, Kelowna, British Columbia V1V 1V7, Canada

**Keywords:** Puma concolor, mountain lion, diet, prey selection, niche theory, spatial overlap, home range

## Abstract

Individual dietary variation in sexually dimorphic predator populations arises from phenotypic traits, preference, prey availability, and competition. We tested competing hypotheses for how diet similarity among cougars (*Puma concolor*) is influenced by sex, seasonal prey availability, and home range overlap in the southern interior of British Columbia, Canada. We proposed 4 hypotheses: (i) prey availability—overlapping individuals share similar diets due to access to the same prey base; (ii) resource competition—overlapping individuals diverge in diet to minimize competition; (iii) resource partitioning—diet similarity peaks at intermediate overlap; and (iv) null—sex and season explain similarity irrespective of overlap. We analyzed diet data from 27 female and 11 male cougars (875 kill sites) and extrapolated prey availability from 146 remote cameras to test for sex-based differences in prey selection across winter, migration, and summer. We then used a pairwise approach to model diet similarity between individuals. Males generally killed larger prey (eg, moose, elk), while females killed more deer, with differences in deer species selection in the Boundary study area where fewer large prey were available. We found support for resource partitioning between female–female dyads and for resource competition between male–female dyads. Diet similarity was lowest in summer when home ranges were largest and highest in winter when ranges were smallest. Our findings highlight the complexity of predation patterns and the roles of sexual dimorphism, seasonal prey availability, and resource partitioning in shaping prey use among spatially overlapping territorial predators in multiprey systems.

## Teaser

We found that, in cougars, dividing prey by species and size facilitates co-existence between these territorial predators. Cougar males are larger than females, spatially overlap in their territories, and are documented consuming different prey species. We showed that male and female cougars that share the same space at the same time have less similar diets than adjacent, non-overlapping cougars in all seasons.

## Introduction

Ecological niche theory relates individuals' fitness to their environment, encompassing the conditions and resources necessary for survival and reproduction (Van Valen 1965). Generalist species maintain wider ecological niches relative to specialist species, though individuals within a generalist population can specialize in their resource use (Pianka 1973). The mechanisms behind individual resource use are grounded in Optimal Foraging Theory (Schoener 1971), where individuals choose among available resources to maximize their net energy gain and promote reproductive success. Individual variation in resource use can arise from genetic differences (Araújo et al. 2011), phenotypic plasticity (Schoener 1986), and environmental heterogeneity (Bolnick et al. 2007). If resource availability is high, individuals may coexist with little niche differentiation (Araújo et al. 2011). In contrast, when individuals occur in habitats with shared and limited resources, they may adjust their resource use, behavior, or habitat selection to minimize competition with conspecifics (Schoener 1986; Araújo et al. 2011).

Resource partitioning is a mechanism by which species, or individuals within a species, reduce the effects of competition through the division of food resources or the spatial or temporal segregation of resource use (Schoener 1974; Bolnick et al. 2003; Pringle 2021). For example, different species of seabird partition water depth for access to resources (Cody 1973), and individuals of *Anolis (spp.)* lizards partition microhabitat and consume different insect species (Schoener 1974). Temporal resource partitioning can be linked to traits that provide competitive advantages; for example, larger bodied birds consume carcasses in the Serengeti earlier in the day than smaller bodied birds (Handler et al. 2021). The partitioning of resources by individuals can lead to prey selection, in which individuals disproportionately use a resource relative to its availability (Backwell et al. 1998; Hayward and Kerley 2005), and within-population dietary specialization, in which individuals use a subset of the resources used by the population (Van Valen 1965; Schoener 1974). Dietary specialization is thought to increase population niche breadth and stability and ultimately facilitate range expansion (Lanszki et al. 2022) and speciation (Chesson 2000). In sexually dimorphic species, resource partitioning may arise by sexual size dimorphism (Bolnick et al. 2003; Meiri et al. 2014), where sexes of different sizes consume different sized prey to ensure prey profitability is maximized in the population as observed in mink (*Mustela vision*; Birks and Dunstone 1985) and African lion (*Panthera leo*; Funston et al. 1998). Sexes may further diverge in prey use in association with seasonal reproductive demands, and behavior (Ruckstuhl and Neuhaus 2005; Thiemann et al. 2011; Ratcliffe et al. 2013).

Cougars (*Puma concolor*) are an example of a sexually dimorphic, generalist predator with the potential to specialize on prey (Knopff et al. 2010). Cougars are one of the most wide-ranging large carnivores in the Western Hemisphere and have been documented killing 232 different prey species (Karandikar et al. 2022). They are sexually dimorphic, with males weighing 36 to 90 kg whereas females weigh 29 to 64 kg (Hall 1981;Nowak 1991). Males overlap and breed with 3 to 5 females within their home range (Logan and Sweanor 2001; Sunquist and Sunquist 2002). Cougar populations are regulated by density dependence and exhibit mutual avoidance (Seidensticker et al. 1973; Elbroch et al. 2016) and intraspecific killing (Seidensticker et al. 1973). Both male and female cougars avoid and kill members of their own sex (Maletzke et al. 2014; Elbroch et al. 2016), though do overlap to some extent with same-sex individuals (Elbroch et al. 2015).

Spatial overlap between individuals in cougar populations has been explored in the context of kinship and hunting habitat availability (Elbroch et al. 2015), though not with respect to diet similarity and prey selection. Cougars have well documented, sex-specific use, with males generally killing larger ungulates like moose (*Alces alces*) and male elk (*Cervus canadensis*; Ross and Jalkotzy 1992; Knopff et al. 2010) while females tend to kill deer and young ungulates. However, in some systems, adult female cougars also kill elk and feral horse (*Equus caballus*; Kortello et al. 2007; Knopff et al. 2010; Davidson et al. 2014; Andreason et al. 2021), suggesting that female diets are not size limited relative to males. The seasonal ungulate birth pulse benefits both male and female cougars as they increase their predation rates on small-bodied prey in the summer months (Knopff et al. 2010; Davidson et al. 2014). Knopff et al. (2010) found specialist tendencies in male and female cougars of all age-classes in a population in Alberta, Canada, and suggests that specialization is more common than previously considered. Dietary specialization in cougars has been hypothesized to be an opportunistic or learned behavior (Ross et al. 1997; Festa-Bianchet et al. 2006), though it may arise as a strategy to alleviate intra-specific resource competition, particularly between males and females (Lowrey et al. 2016). Understanding the mechanisms by which individual variation in resource use occurs can help guide wildlife conservation and management initiatives where cougars target high value prey, such as livestock (Elbroch and Wittmer 2013) or endangered species (Bird et al. 2010; Apps et al. 2013).

Here, we examine seasonal, sex-based differences in prey composition (hereafter, “diet’) in a population of cougars overlapping in space across the southern interior of British Columbia, Canada. First, we tested for seasonal differences in prey biomass killed and prey species selection between male and female cougars. We then tested alternative hypotheses for how spatial overlap influences diet similarity, measured as prey species composition and biomass, among pairs of same- or opposite-sex cougars (dyads, sensu Elbroch et al. 2016), as follows: (i) *prey availability hypothesis*, where peak diet similarity occurs when individuals overlap compared with distant individuals, as shared environments provide access to the same prey base; (ii) *resource competition hypothesis*, where competition for resources in overlapping ranges leads to lower diet similarity compared with nonoverlapping individuals; (iii) *resource partitioning hypothesis,* where peak diet similarity occurs for adjacent ranges with little to no overlap and individuals access similar prey without strong competition; and (iv) *null hypothesis*, where diet similarity is driven primarily by sex and seasonal differences, irrespective of spatial overlap. We predicted that diet similarity would be greatest between same sex cougars, which are more comparable in body size and overlap less extensively than opposite sex dyads (Ross and Jalkotzy 1992; Knopff et al. 2010; Lehman et al. 2017) and that diet similarity would vary seasonally with lower similarity during summer when resources diversify and neonate ungulates become available (Asher 2011; Wright 2024).

## Methods

### Study areas

**Figure 1:**
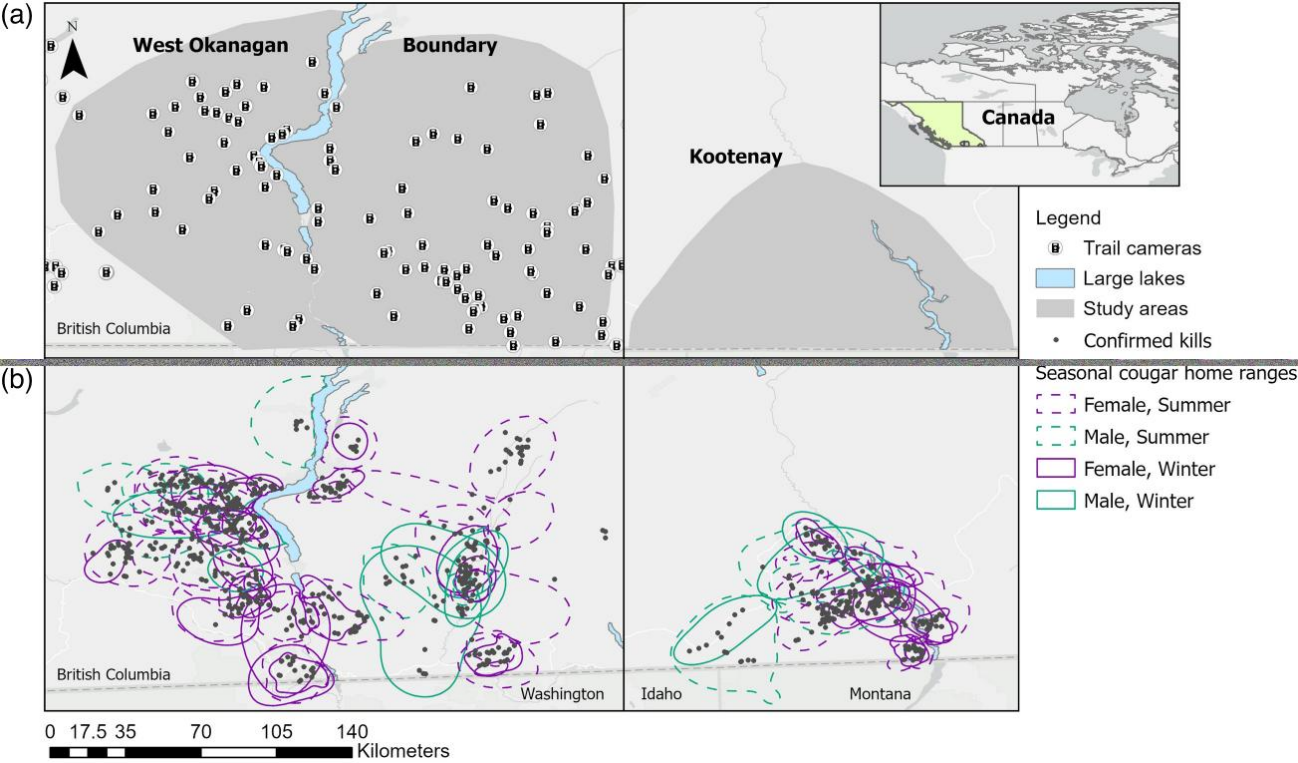
a) Distribution of 146 remote cameras deployed within the West Okanagan and Boundary study areas from 2019 to 2022. Cameras were deployed in winter and summer, and mean ungulate detections were interpolated across summer and winter cougar home ranges using Ordinary Kriging. Values for prey availability during the mule deer migration were averaged between winter and summer. b) Distribution of confirmed kill sites (*n* = 875) in Autocorrelated Kernel Density Estimated seasonal cougar home ranges for GPS-collared males (*n* = 11) and females (*n* = 27) during winter and summer seasons in the southern interior of British Columbia from February 2020 to May 2023. Light Gray Canvas Map [basemap] Sources: Esri, DeLorme, HERE, MapmyIndia.

Our study was in 3 areas in the southern interior of British Columbia, Canada (Fig. 1) in parts of the traditional territories of the Syilx Okanagan, Sinixt, Secwépemc, and Ktunaxa Nations. The West Okanagan study area (5,800 km^2^) extends from Princeton to the west side of Okanagan Lake and from Kelowna south to the United States border. The Boundary study area (5,200 km^2^) extends from Vernon south to the United States border. The Kootenay study area (3,800 km^2^) extends from Yahk to Newgate and from Cranbrook south to the United States border. These areas were selected as representative areas that differ in predator and prey communities, and they overlapped with existing research projects that included a remote camera network established to monitor southern B.C. mule deer populations. The primary ungulate species include mule deer (*Odocoileus hemionus*), white-tailed deer (*Odocoileus virginianus*), moose, and elk with lower abundances of California bighorn sheep (*Ovis canadensis*), mountain goat (*Oreamnos americanus*), feral horse, and domestic livestock. Cougars share the landscape with other interspecific competitors, including gray wolf (*Canis lupus*), coyote (*Canis latrans*), black bear (*Ursus americanus*), grizzly bear (*Ursus arctos*), Canada lynx (*Lynx canadensis*), and bobcat (*Lynx rufus*).

### Cougar GPS collaring and cluster site investigations

We fitted Global Positioning System (GPS) collars (Model: GPS-Plus, Vectronic Aerospace GmbH, Berlin, Germany) on male and female cougars between December 2019 and May 2023. We located cougars by identifying fresh tracks in the snow and used trained, GPS-collared hounds to pursue and tree individuals (Logan et al. 1986; McBride et al. 2008). We chemically immobilized cougars using Telazol (Harthoorn 1976). We aged cougars in the field using gumline recession and tooth staining (Laundré et al. 2000), identified sex using anatomical features, and measured body length and mass (Figure S1). We conducted cougar capture and handling following University of British Columbia Animal Care Protocol A19–0276 and provincial *Wildlife Act* permit MRPE 19-596043. We programed GPS collars with 2-h fix rates and locations updated daily.

### Prey composition

We used cluster analysis (Anderson and Lindzey 2003) to identify potential kill sites from GPS data. In order to increase the probability of locating depredated animals, we defined clusters as those locations with a least 5 GPS locations (8 h minimum duration) within a 100 m radius over 3 days (Knopff et al. 2010). Between February 2020 and August 2023, we searched for remains at clusters using circular transects to 100 m in radius (Anderson and Lindzey 2003), and where possible, identified prey species and sex using anatomical and pelage features (Stelfox 1993). We submitted hair samples from unidentified deer species to be genotyped by Wildlife Genetics International (WGI, Nelson, BC, Canada) using 3 microsatellite DNA loci to distinguish between mule deer and white-tailed deer (Anderson et al. 2002; Miller et al. 2019). We submitted ungulate incisor tooth samples to Matson's Lab (Matson's Laboratory, Milltown, MT, USA) to be aged using cementum annuli analysis (Sergeant and Pimlott 1959).

We investigated kill sites as early as possible to collect reliable prey samples; however, we could not reliably measure prey biomass in the field as carcasses were partially or entirely consumed upon discovery. We therefore classified prey biomass based on their live weight range by species, age, and sex from published reports in British Columbia and other literature (1 to 20 kg, 21 to 50 kg, 51 to 75 kg, 76 to 120 kg, > 120 kg; Table S1) and binned data into 5 categories following natural breaks. We further classified prey species into 5 categories as elk, moose, mule deer, white-tailed deer, and all other prey combined. We were unable to identify species from genetic analysis for 47 deer hair samples and excluded these kills from further analysis. We compared the proportion of kills between males and females for each prey species category within each study area and season, and for prey biomass categories across all study areas combined, using Fisher's exact test. For both prey composition and biomass analyses, we considered *P*-values <0.05 as significant and applied a conservative Bonferroni correction of <0.01, calculated by dividing the alpha value by the number of categories (*n* = 5) to account for multiple comparisons (Wright 1992).

### Seasonal home range estimation

We quantified the 95% seasonal home range kernels for each cougar using Autocorrelated Kernel Density Estimators (AKDE) from GPS collar data in continuous-time movement models (Fleming et al. 2014) in the R package “ctmm” version 1.2.0 (R version 4.3.3; Calabrese et al. 2018; R Core Team 2023). We estimated AKDEs for winter (November 15 to March 15), summer (May 15 to September 15) and the periods in between as the combined seasonal migration for large ungulates (September 16 to November 14 and March 16 to May 14; Poole and Mowat 2005; Mulligan 2021; Wright 2024). We used major lakes > 1 km in width in the Okanagan Valley, which do not freeze during winter, as barriers in the AKDE models to ensure only accessible areas were included in the home range estimates (Hollins et al. 2025). We tested for seasonal differences in home range size for each sex using linear mixed-effects models with cougar ID as a random effect and performed Type III ANOVA with Satterthwaite's approximation (Kuznetsova et al. 2017).

### Prey availability in the West Okanagan and boundary

We obtained ungulate detection data from a concurrent research study between 2019 and 2022 in the West Okanagan and Boundary study areas (Foster 2025; Fig. 1) to interpolate relative prey availability within cougar home ranges. A total of 146 remote cameras were deployed in a stratified-random design for multispecies detection, with summer grids active April–September and winter grids active October–March. Cameras were positioned 4 to 6 m from game trails or dirt roads at ∼1 m height, and vegetation within the detection area was cleared during setup and maintenance to maximize detection. Three camera models were used (Reconyx PC900 Hyperfire 1 and 2 Professional Covert infrared, and Bushnell Trophy Cam HD Aggressor), each set to high-sensitivity motion capture in 3-photo burst mode with a 1-s delay.

We recorded 1,771 images of elk, 2,704 images of moose, 55,441 images of mule deer and 27,147 images of white-tailed deer captured on cameras deployed for 88 ± 48 days (Foster 2025; Table S2). Images were initially classified by a team of 200 trained citizen scientists using Wildlife Insights (Ahumada et al. 2020) and then verified by 2 experienced technicians. We pooled data across years and used Ordinary Kriging (Krige 1951; Wackernagel 1995) to interpolate the mean detection frequency (number of detections per camera per day) of each ungulate prey species for winter and summer camera detections and used the mean values for both seasons as a proxy for spring and fall migration (Table S2). While mule deer are long-distance migrators in this system, their migration pathways are largely captured within the study area boundaries (Wright 2024); therefore, we assume that this approach reasonably represents mule deer detection frequency. Ordinary Kriging estimates mean values in unsampled locations and accounts for spatial autocorrelation in the distribution of the data (Kravchenko 2003; Li and Heap 2011). We used zonal statistics in ArcMap 10.8.1 to obtain estimates within the 95% seasonal cougar home range AKDE boundaries.

### Prey selection between male and female cougars

We measured cougars' selection for the 4 most common species of ungulate prey observed at kill sites (elk, moose, mule deer, and white-tailed deer) using Jacobs' selection index D (Jacobs 1974) in the R package dietr (R version 4.3.3; Borstein 2020; R Core Team 2023). Jacobs' D was calculated for separately for the West Okanagan and Boundary study areas, where each prey species *i* for each individual cougar *j* during a given season (*D_ij_*), where:


$$D_{ij}=(a_{ij}-b_{ij})/(a_{ij}+b_{ij}-2a_{ij}b_{ij}),$$

with *a_ij_* being the proportion of prey species *i* at kill sites for cougar *j*, and *b_ij_* being the proportion of available prey species *i* within the seasonal home range of cougar *j*, derived from interpolated camera data collected between 2019 and 2022 (Table S2). We then summarized *D* values across individuals to obtain mean values and 95% confidence intervals for each sex and study area. Values of D range from −1 (avoidance) to 1 (preference), with intervals overlapping 0 indicating proportional consumption relative to availability. Confidence intervals were estimated using nonparametric bootstrapping (10,000 replicates) to account for small sample sizes and non-normal distributions of selection values (Efron and Tibshirani 1993).

### Between-individual diet similarity

We used diet similarity as a response variable to test for resource partitioning in cougars. We estimated the proportional diet similarity between individuals using the “overlap” function in the R package “RInSp” (Zaccarelli et al. 2013). This function calculates the pairwise diet overlap between all individuals in a sampled population based on their prey frequencies (Bolnick et al. 2003). We measured diet composition for each individual during winter, seasonal migration and summer as 5 classes of prey observed at cluster sites, including elk, moose, mule deer, white-tailed deer and other low frequency prey combined (Table 1) and further classified prey by biomass to account for size differences across prey age classes (Table S1). We limited our analysis to a minimum of 5 prey observations per individual-season to ensure diet was reasonably represented. We then randomly sampled a maximum of 10 prey observations per individual-season to limit mismatches in sampling effort across individuals.

**Table 1:** Predictor variables included in generalized linear mixed-effect models for predicting DSi trends between home range centroids of cougar dyads.

Covariate names	Description
DSi	Response variable: Diet Similarity index value between 0 and 1 calculated from confirmed predation events of ungulate prey species or other prey combined, and biomass.
Bcoeff: Low, Intermediate, High	Bhattacharyya coefficient, estimated from home range centroid distances and size obtained from the 95% AKDE, between pairs of individuals as a measure of home range overlap derived using the “ctmm” R package. Values are categorized using natural breaks reflecting distant, nonoverlapping home ranges (Low), adjacent and minimally overlapping home ranges (Intermediate), and extensive home range overlap (High).
Season: Winter Migration Summer	Time of year classified as winter (November 15 to March 15), summer (May 15 to September 15), or migration corresponding to the spring and fall mule deer migration (September 16 to November 14 and March 16 to May 14).
Dyad: M-F F-F	The sexes of 2 individuals as Female-Female, Male-Female or Male-Male.
CID	Random effect of individual cougars.

To measure diet similarity between *N* individuals, we generated an *N* × *N* matrix, where each cell *o_ik_* represents the diet overlap between individual *i* and individual *k*. The overlap values ranged from 0, when individuals have no common prey, to 1, when individuals kill the same prey in identical proportions. The individual overlap was:


$$\mathrm{Dsi}=o_{ik\;=\;}\sum_{j}\min\left(\;p_{ij},\;p_{kj}\right)$$

where *p_ij_* and *p_kj_* are the proportion of the resource *j* for the 2 individuals.

### Home range overlap

To measure spatial home range overlap between pairs of cougars, we calculated the Bhattacharyya coefficient (Bhattacharyya 1943) using the “overlap” function in the R package “ctmm” (Winner et al. 2018). The Bhattacharyya coefficient (Bcoeff) measures the volumetric overlap between the 95% AKDE home ranges while accounting for the distance between home range centroids where values range from 0 for distant and different sized ranges to 1 for complete overlap of similar sized ranges (Fieberg and Kochanny 2005). We categorized Bcoeff into 3 groups: low (< 0.1), intermediate (0.1 to 0.4), and high (>0.4) to test for differences in diet similarity between different degrees of home range distance, size, and overlap between individuals.

### Resource partitioning analyses

We tested competing hypotheses regarding the drivers of diet similarity among cougars, including effects of prey availability (peak diet similarity at low home range overlap), resource competition (peak diet similarity at high overlap), and their interaction (peak diet similarity at intermediate overlap). To quantify these predictions, we used generalized linear mixed-effects models (GLMMs; Ferrari and Cribari-Neto 2004; Brooks et al. 2017) with a beta family and logit link to model diet similarity as a function of home range overlap (Bhattacharyya coefficient categorized), dyad, and season. We included the interaction between overlap and dyad to test whether diet similarity differed between same-sex and opposite-sex dyads. Pairs of individuals that were not monitored concurrently or did not occur in the same study areas were excluded. We further tested whether diet similarity is best predicted without home range overlap accounted for (season and sex only), and a statistical null model. Individual Cougar ID (CID) was included as a random effect in all models to account for repeated measures, where each individual could overlap with multiple others. We completed model selection using function “model.sel” in the R package “MuMIn” (Barton 2024) and ranked models by AICc weight (Burnham and Anderson 2002).

## Results

### Seasonal diet composition and home range size

We GPS-collared 38 adult cougars (27 females, 11 males) from December 2019 to May 2023 (Fig. 1). Cougar home ranges varied across seasons and sexes (Table S3). Female home ranges differed significantly across seasons, increasing from winter (296 ± 221 km^2^) to migration (441 ± 230 km^2^) and summer (450 ± 220 km^2^; *P* < 0.001). Male home ranges did not differ significantly across seasons, averaging 567 ± 291 km^2^ in winter, 490 ± 254 km^2^ during migration, and 694 ± 399 km^2^ in summer (*P* = 0.20).

**Figure 2:**
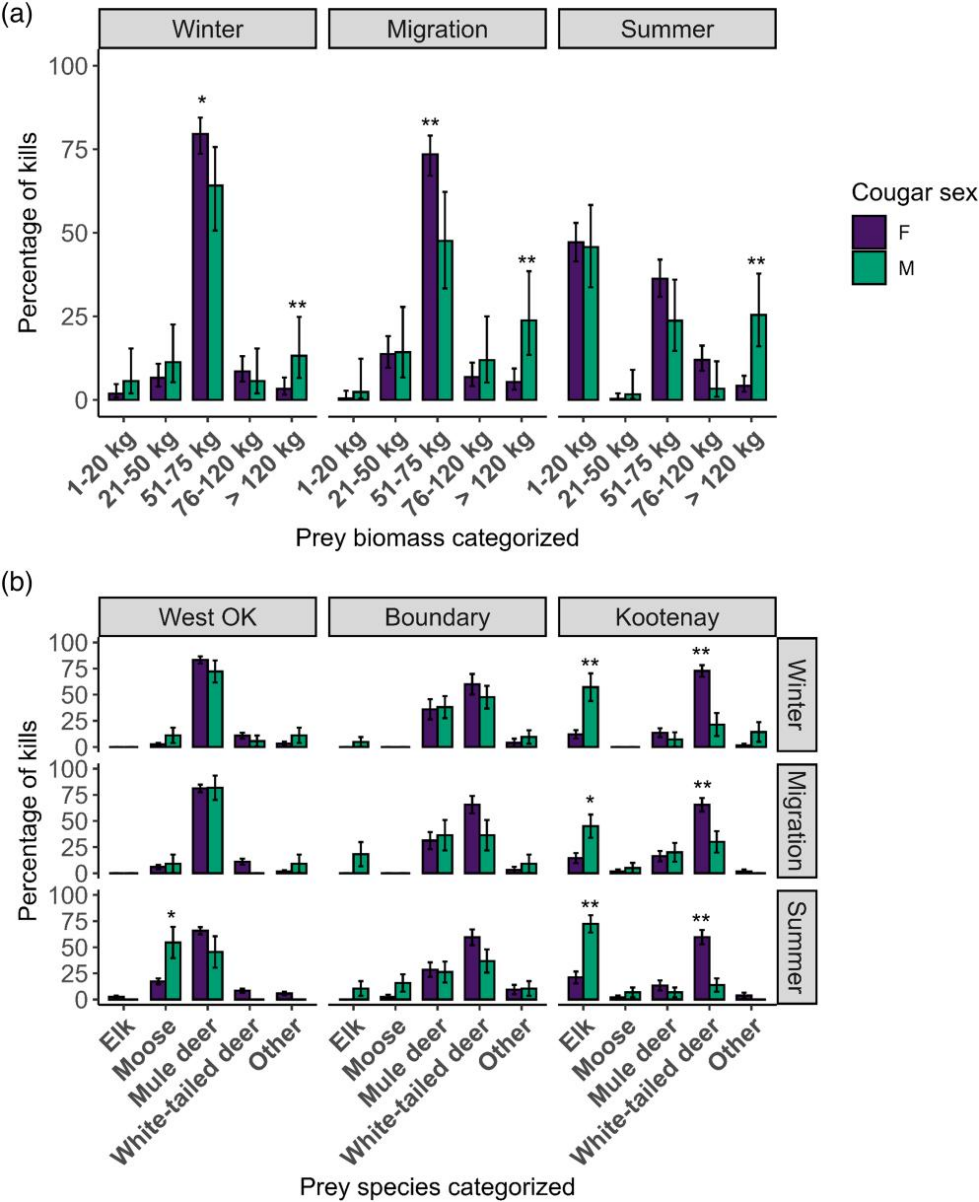
Cougar diet composition by sex, season, and study area from January 2020 to August 2023. Panel a) Proportion of cougar kills by biomass (kg) for female (F) and male (M) cougars by season, pooled across study areas. Biomass classifications are based on species- and age-specific values from the literature (Table S1). Panel b) Proportion of cougar kills by species for female and male cougars across study areas and seasons. Statistically significant sex differences were assessed with Fisher's exact tests; asterisks denote significance at *P* < 0.05 (*) or Bonferroni-corrected *P* < 0.01 (**; *n* = 5 prey groups).

We investigated GPS clusters a mean of 7.4 ± 24 days after formation, confirming 875 kills of 14 prey species at 1,385 cluster sites (Table 2). Using the Fisher's Exact Test, we found that prey composition differed by sex and season within each study area (Fig. 2a, Table S4). Significant sex differences were most pronounced in the Kootenay area. In winter, males killed 57.1% elk (*P* = 0.001) and females killed 72.7% white-tailed deer (*P* = 5.24 × 10^−4^). During migration, males killed 45.0% elk (*P* = 0.011) and females killed 65.5% white-tailed deer (*P* = 0.009). In summer, males killed 72.4% elk (*P* = 1.11 × 10^−5^) and females killed 59.6% white-tailed deer (*P* = 6.03 × 10^−5^). No significant sex-based differences were observed in the West Okanagan or Boundary areas.

**Table 2:** Frequency of confirmed cougar kills located during cluster site investigations by prey species, study area, and season from February 2020 to August 2023.

Species	West Okanagan			Boundary			Kootenay			Total
	Mig	Win	Sum	Mig	Win	Sum	Mig	Win	Sum	
Elk	0	0	5	2	1	2	16	16	32	**74**
Moose	6	5	39	0	0	4	2	0	3	**59**
Mule deer	98	112	122	14	17	17	13	11	9	**413**
White-tailed deer	13	14	16	23	25	31	40	51	35	**248**
Deer sp.	1	4	13	5	5	8	0	1	10	**47**
Other prey:	2	5	8	1	3	4	2	2	4	**31**
Beaver	0	0	0	0	0	1	1	0	0	**2**
Bobcat	0	0	1	0	0	0	0	0	0	**1**
Cougar	0	0	0	0	0	0	0	1	1	**2**
Coyote	0	2	1	0	1	2	0	1	0	**7**
Domestic cat	0	0	1	0	0	0	0	0	1	**2**
Domestic cow	0	1	0	0	0	0	0	0	0	**1**
Porcupine	0	0	1	0	0	0	0	0	0	**1**
Raccoon	0	1	1	0	0	0	0	0	0	**2**
Red squirrel	0	0	1	0	0	0	0	0	0	**1**
Snowshoe hare	0	0	1	1	1	0	1	0	1	**5**
Unknown	2	2	3	0	1	1	0	0	1	**10**
Total confirmed kills	120	141	206	45	51	66	72	81	93	**875**
Total clusters visited	185	238	310	61	68	111	109	146	157	**1,385**

Values in bold are totaled across study areas and seasons. Mig = migration, Win = winter, and Sum = summer. Unidentified deer were classified as “Deer sp.” Prey species outside the 4 primary ungulate species were grouped as “other prey”.

Prey biomass, pooled across study areas, also differed by sex (Fig. 2b; Table S5). In winter, males killed more large prey (>120 kg; 13.2% vs. 3.2%, *P* = 6.44 × 10^−4^), while females killed more intermediate prey (51 to 75 kg; 80.5% vs. 64.1%, *P* = 0.028). During migration, males again killed more large prey (21.7% vs. 5.1%, *P* = 0.001), whereas females killed more intermediate prey (73.9% vs. 50.0%, *P* = 0.002). In summer, males took substantially more large prey (24.2% vs. 3.9%, *P* = 2.26 × 10^−6^), but both sexes killed small prey (1 to 20 kg) at nearly equal rates (46.8% vs. 47.9%, *P* = 0.887). These patterns indicate that males relied more on large-bodied prey across all seasons, whereas females targeted intermediate prey in winter and migration, with both sexes relying heavily on small prey in summer.

### Prey selection in the West Okanagan and boundary

**Figure 3:**
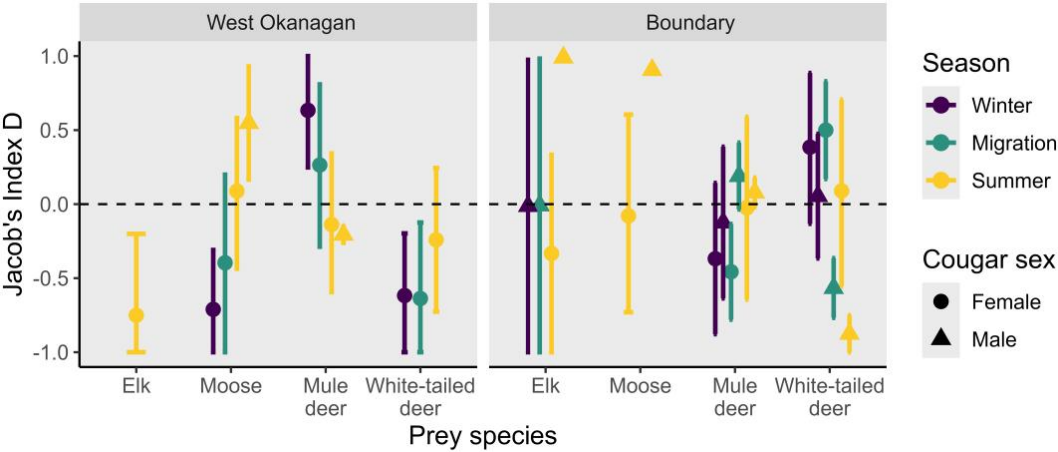
Jacob's index D and 95% confidence intervals for seasonal ungulate prey selection by female and male cougars in the West Okanagan and Boundary study areas combined. The Kootenay study area is excluded here due to limited prey availability data. Values overlapping 0 indicate proportional predation to availability, positive values indicate selection, and negative values indicate avoidance. Data were insufficient to estimate selection of elk by West Okanagan male cougars in summer.

Cougars differed in their prey selection patterns by sex, season, and study area. Females in the West Okanagan strongly selected for mule deer ([mean *D*] = 0.63, [95% CI] = 0.51 to 0.76) while avoiding moose (−0.71, −0.83 to −0.58) and white-tailed deer (−0.61, −0.75 to −0.48) in winter with similar trends during migration (Fig. 3). Conversely, females in the Boundary study area avoided mule deer (−0.37, −0.58 to −0.15) and selected for white-tailed deer (0.38, 0.16 to 0.60) in winter with similar patterns during migration. Females in both study areas killed all prey proportionately to their availability except for elk during summer (Fig. 3), where males strongly selected for moose in the West Okanagan (0.55, 0.16 to 0.93) and in the Boundary (0.90, 0.89 to 0.92). Males killed mule deer in the Boundary proportionately to their availability in all seasons and avoided white-tailed deer during migration and summer (Fig. 3). Data were deficient for West Okanagan males during winter and migration to estimate confidence intervals for prey selection (Table 2).

### Between-individual diet similarity

**Figure 4:**
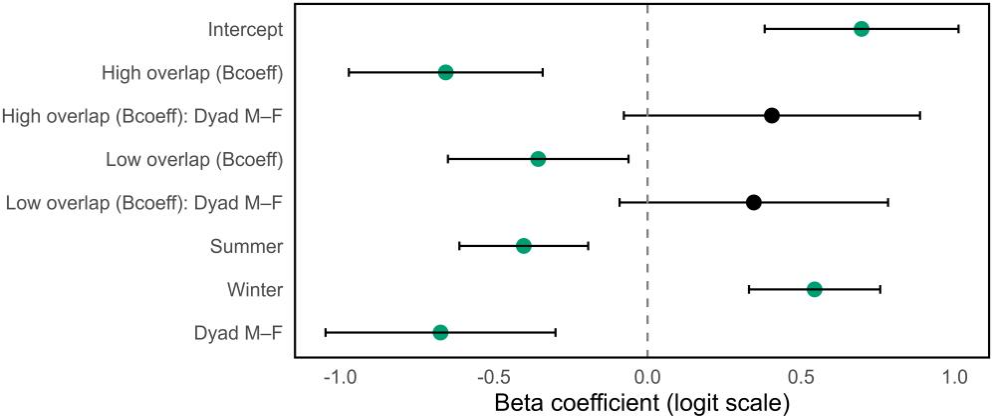
Beta coefficients and 95% confidence intervals for the top generalized mixed-effects model (logit link) testing the effects of home range overlap (Bhattacharyya coefficient classified as low, intermediate, high), dyad type (male-female, female-female), and season (winter, migration, summer) on individual diet similarity. The vertical grey line at zero indicates no effect relative to the reference categories (F–F dyad, low overlap, migration season). Data are pooled across study areas and years from January 2020 to August 2023. Data were deficient for male-male dyads.

**Figure 5:**
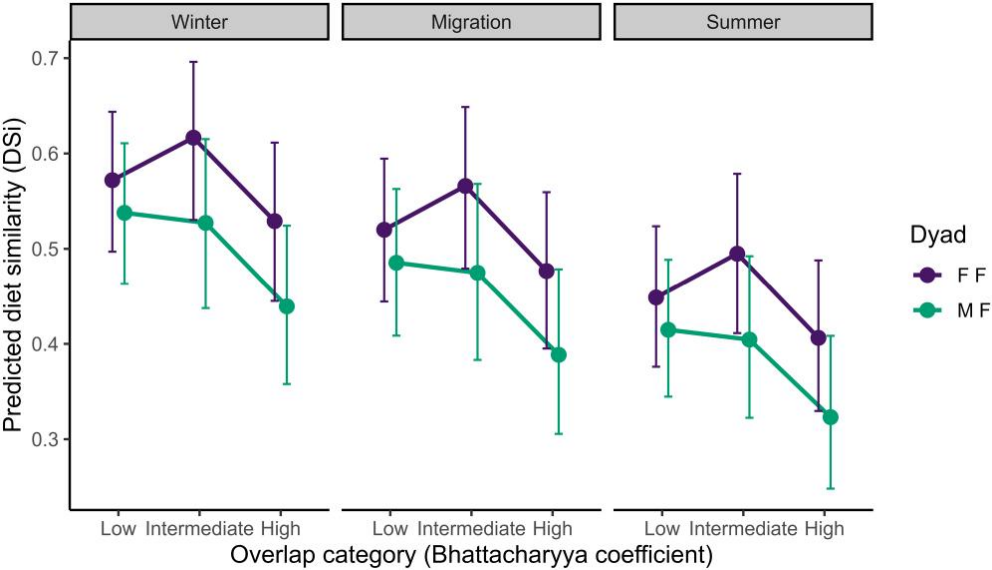
Predicted diet similarity (DSi) and 95% confidence intervals for cougar dyads across overlap categories and seasons. Values represent model-predicted means (±95% CI) from a beta regression of diet similarity as a function of home range overlap (Bhattacharyya coefficient as low, intermediate, high), sex pair (F–F, M–F), and season (winter, migration, summer). Predictions are shown separately for each season, with purple lines indicating female–female dyads and teal lines indicating male–female dyads. Data are pooled across study areas and years.

We used a mean of 6.4 ± 3.2 kills per individual per season in diet similarity (DSi) calculations, observing 12 total prey groups (prey species × biomass). We obtained sufficient data for female-female and male-female dyads to test our hypotheses on diet similarity across space ([*n*] Winter _F-F_ = 66, _M-F_ = 51; Migration _F-F_ = 54, _M-F_ = 24; Summer _F-F_ = 75, _M-F_ = 40). Diet similarity between cougar dyads was best explained by the home range overlap model (AIC weight = 0.98; Table 3). In female-female dyads, diet similarity was significantly lower at both low overlap (*β* = −0.36, *P* = 0.017) and high overlap (*β* = −0.66, *P* < 0.001) relative to intermediate (Fig. 4), consistent with predictions for resource partitioning (Fig. 5). Male–female diets were significantly less similar overall than the default (*β* = −0.67, *P* < 0.001), with comparable similarity at low and intermediate overlap, and significantly reduced diet similarity at high spatial overlap (Fig. 4), following predictions for resource competition (Fig. 5). Diet similarity varied seasonally, being highest in winter (*β* = 0.54, *P* < 0.001) and lowest in summer (*β* = −0.40, *P* < 0.001), relative to migration. These results indicate that prey use is least similar when cougars exhibit high spatial overlap, particularly in male–female dyads, and that seasonal shifts further shape the extent of diet similarity (Fig. 5).

**Table 3:** Generalized linear mixed-effects models predicting diet similarity (DSi) among GPS-collared cougars as a function of home range overlap, dyad type, and season.

Model name	Formula	df	LogLik	AICc	ΔAIC	Weight
Home range overlap effects	DSi ∼ Bcoeff: Dyad + Dyad + Season + (1|CID)	10	72.61	−124.30	0.00	0.98
Season and sex	DSi ∼ Season + Dyad + (1|CID)	6	64.48	−116.60	7.70	0.02
Null	DSi ∼ 1 + (1|CID)	3	54.14	−102.20	22.13	0.00

Home range overlap was quantified using Bhattacharyya's coefficient (Bcoeff). Dyad type refers to sex-based cougar pairs. Seasons included winter, migration, and summer. Models included a random intercept for individual cougars (CID). Columns show the number of parameters (df), log-likelihood (logLik), Akaike's Information Criterion corrected for small sample size (AICc), ΔAICc relative to the top-ranked model, and model weight.

The second-ranked model (season and dyad only) had negligible support (AIC weight = 0.02; Table 3). Male–male dyads were data-deficient (fewer than 10 observations with Bcoeff > 0.1) and were excluded from models, though descriptive statistics are provided in Table S6.

## Discussion

Understanding how individuals partition resources within populations is a central problem in ecological niche theory, particularly for territorial predators where spatial overlap does not necessarily imply shared resource use. Using cougars as a model species, we found that diet similarity varied with sex, season, and the degree of home range overlap, consistent with predictions from resource competition and resource partitioning hypotheses. Specifically, diet similarity between male–female dyads declined with increasing overlap, whereas female–female dyads peaked at intermediate overlap, indicating distinct mechanisms mediating intraspecific competition. We found evidence of sexual size dimorphism in prey use, with males consuming a greater proportion of large-bodied prey (eg, moose and elk) across seasons while maintaining larger home ranges, potentially allowing greater access to diverse prey (Balme et al. 2020). These findings align with other studies documenting sex-based differentiation in cougar prey use at the population level (Knopff et al. 2010; White et al. 2011; Elbroch et al. 2013; Andreason et al. 2021; Bernard et al. 2023; Bates-Mundell et al. 2024). Here, we examine the mechanisms underlying these dietary patterns and their implications for understanding resource use and intraspecific competition in territorial carnivores.

Irrespective of sex, cougars with high spatial overlap exhibited significantly lower diet similarity by species and biomass. Dyad type and season alone did not best explain diet similarity; rather, including the extent of overlap improved model performance. Male–female dyads had significantly lower diet similarity than female–female dyads. Their similarity patterns followed our predictions for resource competition, where similarity was lowest at high levels of home range overlap. Female–female dyads exhibited peak similarity at intermediate overlap, providing support for resource partitioning. Neighboring females may reduce competition by minimizing spatial overlap or broadening their prey base. System-level differences in prey density or diversity may also shape the extent to which partitioning arises in cougar populations (Meiri et al. 2014), given that partitioning is 1 mechanism for mediating competition when resources are limited (Bolnick et al. 2003; Araújo et al. 2011). Conversely, if prey are abundant, individuals maintaining high overlap may still rely on the same prey base without strong competitive pressures (Bolnick et al. 2003; Araújo et al. 2011).

We examined prey use and diet similarity across 3 seasons to capture variation in predation as ungulates shifted between winter and summer ranges. During winter, deer concentrate on winter ranges (Armleder et al. 1994; Morrison et al. 2003) and may attract neighboring cougars to prey “hot spots” (López-Bao et al. 2011), as observed in the Greater Yellowstone Ecosystem where females increased overlap during winter (Elbroch et al. 2016). Female cougars in our study killed deer throughout the year and maintained smaller ranges during winter, nearly doubling their range size in summer while diversifying their diets. Diet similarity was lowest in summer, which we attribute to the ungulate birth pulse (Asher 2011; Wright 2024). Small prey comprised nearly half of both male and female kills during summer. Both sexes capitalized on the influx of neonates to meet energetic demands at lower cost and risk of injury (Testa et al. 2000; Knopff et al. 2010; Bates-Mundell et al. 2024), while continuing to differentiate by ungulate species. This response contrasts with patterns observed in coyotes, where diets converged among individuals during pulses of ungulate neonates and other seasonal resources (Jensen et al. 2024). The effects of resource pulses on intraspecific diet similarity may depend on resource diversity and the degree of individual specialization or prey selection, particularly between sexes.

Our findings support broader patterns of individual dietary variation in solitary carnivores. Across taxa, intraspecific competition between sexes is often mediated through partitioning along multiple niche axes. For example, fishers (*Pekania pennanti*) exhibit sex-specific differences in space use where females avoid both male and female conspecifics and males tolerate overlap with females for mating opportunities (Facka and Powell 2021). Resident leopards (*Panthera pardus*) with overlapping home ranges exhibit altitudinal partitioning of predation, effectively maintaining exclusive hunting areas within shared territories (Farhadinia et al. 2020). Temporal and fine-scale spatial partitioning have also been observed in lions, where males and females adjust activity patterns within overlapping ranges to reduce interference competition (Funston et al. 1998). These studies illustrate how individuals can occupy the same landscapes while minimizing competition through differentiation across spatial, temporal, or trophic niche axes. We suggest that prey selection can mediate competition between sexes at a population level, and between individuals at high spatial overlap. Accounting for individual-level dietary differentiation is therefore critical for understanding predator space use and population-level diet composition in behavioral ecology studies.

Prey selection may also reflect factors other than overlap or competition. Individual dietary variation can be shaped by personality (Bolnick et al. 2011), phenotype (Svanbeck and Bolnick 2007; Araújo et al. 2011), or learned behavior (Estes et al. 2003; Gosch et al. 2019). Elbroch et al. (2016) found no support for kinship as a driver of overlap in a nonhunted cougar population, but relatedness could influence diet similarity if hunting strategies are learned maternally (Estes et al. 2003). We did not account for spatial clustering of prey or temporal partitioning of space use, which may facilitate coexistence if individuals use the same areas at different times, as in Iberian lynx (*Lynx pardinus*; López-Bao et al. 2011). However, because cougars typically ambush prey nocturnally (Logan and Sweanor 2001; Hornocker and Negri 2009; Knopff et al. 2010), temporal partitioning seems less likely. Niche partitioning between carnivore species may reduce the need for intraspecific partitioning (Ebenmann and Nilsson 1982). We lacked sufficient predator detections on un-baited camera grids to test this, but recommend future studies consider predator communities when examining intraspecific diet similarity.

An additional consideration for our camera trapping approach is that Jacobs' selection index D relies on estimates of prey availability from detections, which assumes that all ungulate species are equally detectable and that detection scales proportionally with true abundance (Rovero and Marshall 2009). Differences in habitat use, behavior, or camera placement could bias these estimates (Hofmeester et al. 2019; Tanwar et al. 2021), influencing *D* values. We consider these data reasonable approximations of prey availability and prey selection within seasonal cougar home ranges, but caution is warranted when interpreting absolute values. We were unable to test diet similarity hypotheses for male–male dyads due to limited sample size. Males were encountered less often than females, likely due to lower densities and larger territories (Logan and Sweanor 2001; Sunquist and Sunquist 2002). Our analyses included a minimum of 5 kills per individual–season and a maximum of 10 to calculate similarity, representing between 1 and 2 months of diet composition. We used lower thresholds for diet calculations to include as many pairs across seasons as possible; overall higher sampling rates would likely improve diet similarity estimation.

Overall, our study shows that prey differentiation by species and biomass can persist at high spatial overlap, with support for resource competition between male and female cougars and resource partitioning among neighboring females. Recognizing individual dietary variation has implications for conservation biology (Bolnick et al. 2003). Management strategies that target individuals that predate on specific prey may overlook dietary complexity or fail to account for seasonal and community-level dynamics that shape prey selection. More generally, our results reinforce that individual-level variation is a critical driver of intraspecific competition and niche structure across solitary carnivores and likely other territorial or resource-limited species. Further work is needed to clarify the mechanisms mediating intraspecific competition and to evaluate how bottom-up and top-down pressures influence individual prey use.

## Supplementary Material

arag060_Supplementary_Data

## Data Availability

Analyses reported in this article can be reproduced using the data provided by Darlington et al. (2026) on Borealis data repository.
