# Supplementary material for: Seasonal resource partitioning between neighboring cougars in a multiprey system
Source: Behav Ecol. 2026 Jun 4;37(5):arag060. doi: 10.1093/beheco/arag060 (PMC13448344; doi:10.1093/beheco/arag060)
Supplement: Supplementary file 1 [file arag060_supplementary_data.pdf]

Supplementary Materials for

Darlington S, Hodges KE, Gooliaff T, Foster ST, Noonan MJ, Ford AT. 2026. Seasonal resource partitioning between neighboring cougars in a multi-prey system. Behavioral Ecology.

Summary

This file includes data summaries for cougar body size by sex, carcass biomass estimates for ungulate prey by species, age and sex, additional methodological details for estimating prey availability from remote camera data, and detailed data tables from resulting analyses of prey composition, selection, and dietary overlap.

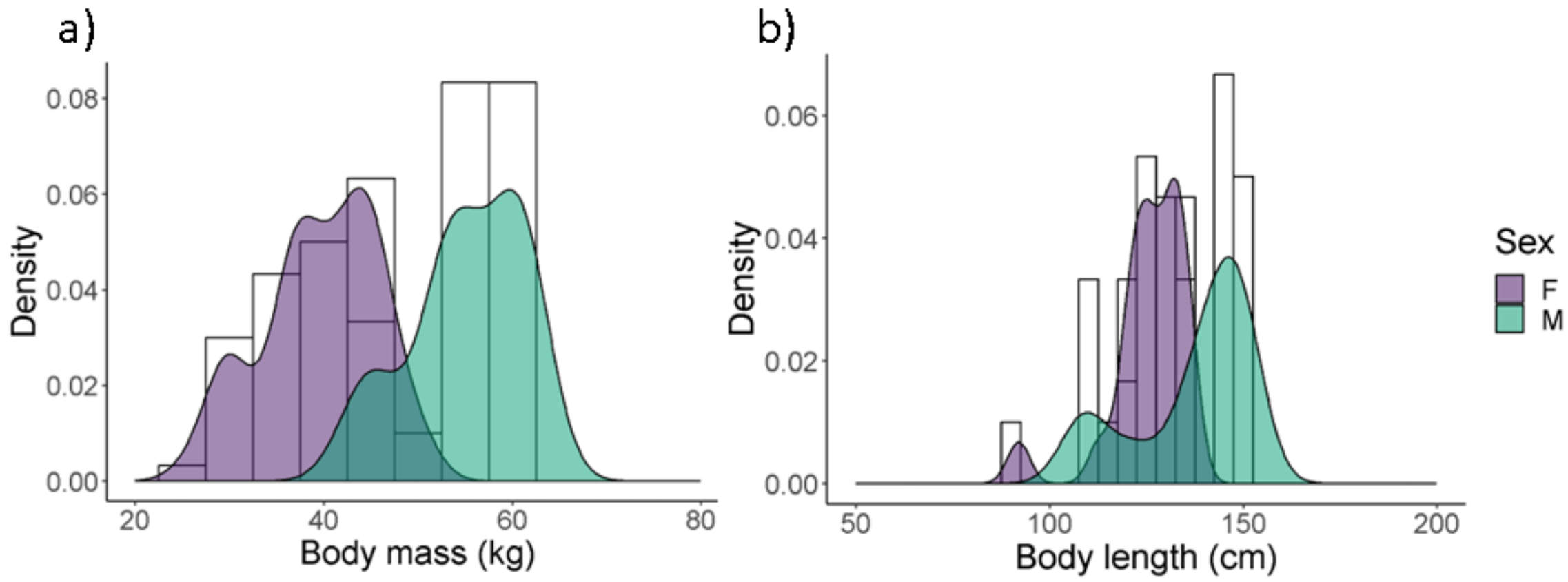

Figure S1. Density distribution for adult female (n=27) and male (n=11) cougar body measurements during initial capture including a) body mass in kilograms and b) body length (tip of nose to base of tail) in cm.

Table S1. Biomass of live prey species in British Columbia by age and sex class as a proxy for biomass killed by male and female cougars located from GPS cluster site investigations. Biomass was classified according to the midpoint value of live weight ranges from literature in the absence of data to estimate the mean and sorted into f bins using natural breaks in the data.

Species	Age class	Sex	Liveweight midpoint (kg)	Live weight range (kg)	Classified biomass (kg)	Source
**Main four ungulates**						
Elk	Neonate	F/M	14	12-16	1-20	BC Ministry of Environment, Lands and Parks 2000a
	6 months	F/M	60	45 - 75	51-75	
	Adult	F	230	190-270	>120	
	Adult	M	337	265-410	>120	
Moose	Neonate	F/M	15	13-18	1-20	BC Ministry of Environment, Lands and Parks 2000b
	6 months	F/M	190	180-200	>120	
	Adult	F	380	340-420	>120	
	Adult	M	475	450-500	>120	
Mule deer	Neonate	F/M	2.5	2-3	1-20	Pettigrew & Jackson, 1980
	6 months	F/M	36	34-38.5	21-50	
	Adult	F	62	50-75	51-75	
	Adult	M	98	68-113	76-120	
White-tailed deer	Neonate	F/M	2.5	2-3	1-20	Pettigrew & Jackson, 1980
	6 months	F/M	29	27-32	21-50	
	Adult	F	59	45 - 73	51-75	
	Adult	M	85	68-102	76-120	
**Other prey**						
Beaver	Adult	F/M	20	11-32	1-20	
Bobcat	Adult	F/M	11	4-18	1-20	
Cougar	Neonate	F/M	0.75	0.5-1	1-20	Kill site data
	6 months	F/M	17	15-20	1-20	
	Adult	F	44	36-53	21-50	
	Adult	M	55	43-67	51-75	
Coyote	Adult	F/M	13	10-16	1-20	
Domestic cattle	Neonate	F/M	35	25-45	21-50	
	6 months	F/M	297	285-310	>120	
	Adult	F/M	727	545-910	>120	
Porcupine	Adult	F/M	11	3.5-18	1-20	Blake & Gese 2016
Raccoon	Adult	F/M	6	3.5 – 9	1-20	

Interpolation of prey availability in the West Okanagan and Boundary

To assess differences in prey availability across space, we deployed 146 remote cameras in a stratified-random design across the West Okanagan and Boundary study areas between 2019 and 2022 (Appendix A, Figure A2). Cameras were not deployed in the Kootenay study area as part of the study. Cameras were set up on summer grids active from April–September and winter grids active from October–March following protocols by Foster (2021). We used 3 camera models, each set to high sensitivity motion capture and 3-photo burst mode with a 1-second delay between photos: Reconyx PC900 Hyperfire 1 and 2 Professional Covert infra-red (Reconyx, WI, USA), and Bushnell Trophy Cam HD Aggressor (Bushnell Outdoor Products, Overland Park, KS). We faced cameras toward game trails and dirt roads to increase large mammal detectability (Cusack et al. 2015). Each camera was set 4-6 m back from the center of a game trail, or roadside, at approximately waist height (1 m). During set-up and annual maintenance visits when cameras were relocated between seasonal grids, we cleared low hanging branches and herbaceous plants to maximize the camera detection area. A team of 200 trained citizen scientists classified images using Wildlife Insights (Ahumada et al. 2020) to identify medium-large mammals species (inclusive of lagomorphs) and number of individuals following procedures by Foster (2021). Following initial image sorting and labeling, each mammal species' image collection was reviewed by two experienced technicians for mislabeled images of similar looking species. The verified image set was used in all analyses. In total, we collected approximately 2.6 million images, with roughly 450,000 containing wildlife. We considered capture events to be independent at 30-minute

intervals to account for pseudo-replication in the data (Ridout and Linkie 2009), and summarized each site and ungulate prey species accounting for active camera days. We averaged camera detections across years for winter and summer grids, and used the mean values for both seasons as a proxy for ungulate detections during fall and spring migration.

We interpolated prey availability across cougar home ranges from 1 771 images of elk, 2 704 images of moose, 55 441 images of mule deer and 27 147 images of white-tailed deer across 146 cameras for 88 ± 48 days. We used Ordinary Kriging (Kridge, 1951) to estimate the average relative detection frequency of each prey species across seasonal cougar home ranges. Ordinary Kriging estimates mean values in unsampled locations and accounts for spatial autocorrelation in the distribution of the data (Kravchenko 2003; Li and Heap 2011).

Table S2. Mean and standard deviation of camera detections of ungulate species by camera day during winter and summer in the Okanagan and Boundary study areas between 2019 and 2022 Values for migration were averaged between winter and summer values for each ungulate species.

Species	Mean detections/camera/day			
	West Okanagan		Boundary	
	Winter	Summer	Winter	Summer
Elk	0.01 ± 0.07	0.005 ± 0.03	0.09 ± 0.65	0.04 ± 0.26
Moose	0.12 ± 0.35	0.47 ± 3.00	0.04 ± 0.16	0.03 ± 0.19
Mule deer	2.97 ± 5.45	2.90 ± 5.72	1.65 ± 3.85	0.86 ± 1.89
White-tailed deer	0.72 ± 3.66	0.15 ± 0.60	1.52 ± 3.72	1.51 ± 4.45
Mean camera days	81.2 ± 65.1	56.2 ± 20.9	106.1 ± 69.2	67.4 ± 25.5

Additional data summaries and reference tables

Table S3. Mean home range size (km^2) by season and sex for 38 GPS-collared cougars (27 females, 11 males) monitored between December 2019 and May 2023 across the West Okanagan, Boundary, and Kootenay study areas in southern interior British Columbia. Reported values are means ± SD. Seasonal effects were tested using linear mixed-effects models with winter as the baseline; p-values are shown for migration and summer relative to winter.

Season	Sex	Home range size km^2 (Mean ± SD)	Anova p-value
Winter	Female	296.1 ± 220.7	(baseline)
	Male	566.8 ± 290.8	(baseline)
Migration	Female	441.1 ±230.2	2.33e-05
	Male	489.9 ± 253.7	0.673
Summer	Female	450.0 ± 220.2	0.00046
	Male	694.2 ± 399.2	0.207

Table S4. Fisher's exact test for differences in species killed by male and female cougars across study areas and seasons. Asterisks reference the p-value threshold for statistical significance, where * satisfies a p-value below 0.05, and ** signifies a p-value below the Bonferroni correction of 0.01 which accounts for the number of prey classes (5).

Season	Prey species	p-value	Mean % male kills	Mean % female kills	Significance
**West Okanagan**					
Winter	Moose	0.127	11.1	2.5	
	Mule deer	0.322	72.2	83.3	
	White-tailed deer	0.694	5.6	10.8	
	Other	0.176	11.1	3.3	
Migration	Moose	0.523	9.1	6.0	
	Mule deer	1.000	81.8	81.2	
	White-tailed deer	0.602	0.0	11.1	
	Other	0.238	9.1	1.7	
Summer	Elk	1.000	0.0	2.6	
	Moose	0.008	54.5	17.4	*
	Mule deer	0.201	45.5	65.8	
	White-tailed deer	0.606	0.0	8.4	
	Other	1.000	0.0	5.8	
**Boundary**				0.0	
Winter	Elk	0.457	4.8	0.0	
	Mule deer	1.000	38.1	36.0	
	White-tailed deer	0.553	47.6	60.0	
	Other	0.585	9.5	4.0	
Migration	Elk	0.061	18.2	0.0	
	Mule deer	1.000	36.4	31.3	
	White-tailed deer	0.156	36.4	65.6	
	Other	0.451	9.1	3.1	
Summer	Elk	0.093	10.5	0.0	
	Moose	0.085	15.8	2.4	
	Mule deer	1.000	26.3	28.6	
	White-tailed deer	0.166	36.8	59.5	
	Other	1.000	10.5	9.5	
**Kootenay**				0.0	
Winter	Elk	7.01E-04	57.1	12.1	**
	Mule deer	0.683	7.1	13.6	
	White-tailed deer	5.24E-04	21.4	72.7	**
	Other	0.078	14.3	1.5	
Migration	Elk	0.011	45.0	14.5	*
	Moose	0.465	5.0	1.8	
	Mule deer	0.737	20.0	16.4	
	White-tailed deer	0.009	30.0	65.5	**
	Other	1.000	0.0	1.8	

Summer	Elk	1.11E-05	72.4	21.2	**
	Moose	0.290	6.9	1.9	
	Mule deer	0.478	6.9	13.5	
	White-tailed deer	6.03E-05	13.8	59.6	**
	Other	0.535	0.0	3.8	

Table S5. Fisher's exact test for differences in biomass killed by male and female cougars across seasons. Asterisks reference the p-value threshold for statistical significance, where * satisfies a p-value below 0.05, and ** signifies a p-value below the Bonferroni correction of 0.01 which accounts for the number of biomass classes (5).

Season and Biomass	Mean % male kills	Mean % female kills	P-value	Significance
**Winter**				
1-20 kg	5.66	1.8	0.147	
21-50 kg	11.3	6.33	0.251	
51-75 kg	64.1	80.5	0.028	*
76-120 kg	5.66	8.14	0.776	
>120 kg	13.2	3.17	6.44E-04	**
**Migration**				
1-20 kg	2.17	0.46	0.313	
21-50 kg	13.0	13.5	1.000	
51-75 kg	50.0	73.9	0.002	**
76-120 kg	13.0	6.97	0.336	
>120 kg	21.7	5.11	0.001	**
**Summer**				
1-20 kg	46.8	47.9	0.887	
21-50 kg	1.61	0.32	0.315	
51-75 kg	24.2	36.33	0.071	
76-120 kg	3.22	11.6	0.060	
>120 kg	24.2	3.85	2.26E-06	**

Table S6. Sample sizes of individual combinations (n total=319) and mean and standard deviation for Diet Similarity Index (DSi) and Bhattacharyya coefficient values by dyad (FF=female-female, M-F= male-female, M-M= male-male) and season (Winter, Migration, Summer).

Dyad - Season		Diet Similarity index		Bhattacharyya coefficient	
	N	Mean	Std	Mean	Std
**Winter**					
F - F	66	0.658	0.175	0.126	0.242
M - F	51	0.489	0.215	0.293	0.366
M - M	5	0.496	0.116	0.633	0.337
**Migration**					
F - F	54	0.582	0.238	0.222	0.300
M - F	24	0.583	0.219	0.231	0.312
M - M	1	0.4	-	0.184	-
**Summer**					
F - F	75	0.527	0.161	0.181	0.261
M - F	40	0.369	0.143	0.124	0.211
M - M	3	0.357	0.156	0.306	0.465

Table S7. Top GLMM model β estimates, standard error, z-value, p-value and significance ($\alpha < 0.05$) for diet similarity (DSi) across individual cougars by dyad, season, and overlap extent as measured by the Bhattacharyya coefficient (low, intermediate, high). Values are compared to default categories for F-F dyad, intermediate overlap, and migration season.

Covariate	β estimate	Std. Error	z-value	Pr(>|z|)	Significance
Intercept	0.697	0.161	4.334	0.000	***
Low Overlap	-0.356	0.150	-2.378	0.017	*
High Overlap	-0.657	0.161	-4.094	0.000	***
Dyad M F	-0.674	0.191	-3.528	0.000	***
Season Summer	-0.403	0.107	-3.757	0.000	***
Season Winter	0.544	0.109	4.974	0.000	***
Low Overlap: Dyad M F	0.346	0.223	1.549	0.121	
High Overlap: Dyad M F	0.405	0.246	1.647	0.100	.
